# How I see is how I feel. Identification of illness perception schema and its association with adaptation outcomes in multiple sclerosis – a 5-year prospective study

**DOI:** 10.1371/journal.pone.0258740

**Published:** 2021-10-28

**Authors:** Jagoda Różycka

**Affiliations:** University of Silesia, Katowice, Poland; Universita degli Studi di Napoli Federico II, ITALY

## Abstract

The aim of the study was to assess the role of illness perception in adaptation to chronic disease among patients with relapsing-remitting multiple sclerosis (RRMS). The differences between the obtained configurations of the illness perception components during four measurements and the model of predictions of the values of adaptation indicators, i.e. depression, anxiety and quality of life during subsequent measurements, were analyzed. Illness representation was assessed at baseline via the Illness Representation Questionnaire–Revised. The adaptation indicators–anxiety, depression (measured by HADS) and quality of life (measured by MSIS-29) were measured at baseline and three more times over a five-year period. The k-means cluster analysis (with two-way and repeated measures ANOVA) was conducted in a group of 90 patients (48.89% women and 51.11% men). Subsequently, the mean values of depression, anxiety, physical and psychological quality of life were compared between the clusters using the Kruskall-Wallis test. Finally, a cross-lagged panel modeled for HADS and MSIS-29 subscales in each measurement occasion (T1-T4). Three different illness perception clusters (Anxious, Realistic and Fatalistic Illness Perception named AIP, RIP and FIP) were composed which differentiated the depression, anxiety, quality of life level and age. FIP showed the lowest adaptation outcomes with small differences between AIP and RIP. It was also significantly characterized by the highest age. The positive adaptation indicators were related to the RIP cluster. The model presented rather satisfactory fit (*χ*^2^(48) = 81.05; CFI = .968; TLI = .925; SRMR = .050) with slightly inflated RMSEA = .087 (90%CI .053-.120). Based on initial measurements of individual characteristics, it was possible to predict the functioning of patients after several years. For patients with AIP, the covariance of anxiety and depression was significant, for patients with RIP–depression and anxiety, and for patients with FIP–depression. In addition, each of the variables was a predictor of subsequent measurements in particular time intervals, illustrating the dynamics of changes. Results highlight that illness perceptions formed at the beginning of RRMS are important for the process of adaptation to the disease. Moreover, they showed the differences between the adaptation outcomes supporting the idea that a cognitive representation might be important for the level of psychological functioning.

## Introduction

Multiple sclerosis (MS) is a progressive neurological disease characterized by an unpredictable course of symptoms such as dizziness and vertigo, vision problems, stiffness, weakness, pain, mobility problems, and cognitive difficulties that contribute to the development of disability [1]. Most often, the disease starts with single relapses and periods of remission of symptoms (the relapsing-remitting type). Consequently, coping with the illness requires numerous psychosocial resources from the patient. The variability as well as the type of multiple sclerosis symptoms is also high–the more symptoms a patient presents while presenting a higher level of disability, the lower the quality of life and the lower the level of coping [2, 3]. The process of adaptation to the disease is demanding in the case of MS due to its unpredictable course, the presence of cognitive impairments, unknown cause, and lack of causal treatment, which makes it difficult to control the disease [4]. The symptoms have a significant impact on the patients’ quality of life (QoL) and emotional functioning (especially on the level of negative emotions) [5, 6]. The quality of life level also affects other areas of life, such as social functioning and life roles [7, 8]. Many studies suggest a lower health related quality of life (HRQoL) in individuals with MS compared to healthy individuals [8–11], as well as to individuals with other chronic conditions such as inflammatory bowel disease, rheumatoid arthritis, epilepsy, diabetes, and cardiovascular disease [12–14]. The quality of life of individuals with MS is related to the degree of disability and the symptoms present [13, 15, 16], among other things. Emotional functioning reflected in the levels of depression and anxiety is also an important factor in the assessment of the adaptation process. Depressive disorders occur in up to 50% of individuals living with multiple sclerosis [17–20]. Depression together with anxiety disorders determine the level of the patient’s functioning [21]. The frequency of such disorders in this group is generally 2–3 times higher compared to the general population. Numerous etiological factors may influence the development of depression and anxiety in MS, including biological mechanisms (e.g., hippocampal microglia activation, comorbid disease burden, CNS damage in various areas), but also the stressors, threats and losses that accompany living with an unpredictable and often permanently disabling disease [22].

Consequently, the factors are sought which might act as a buffer in minimizing the adverse effects of multiple sclerosis. Identifying their importance may have direct application in individual care, development of services, and promotion of new psychological intervention strategies for patients with MS. One such factor is illness perception, understood as a complex and dynamic cognitive schema, bringing together beliefs, knowledge, personal judgements and opinions about the condition. An attempt was made to empirically distinguish in the studied sample of MS patients subgroups composed of individuals who declare/have a similar illness perception.

### Illness perception

How a patient perceives their illness affects the quantity and quality of the efforts they make to change their health situation as much as possible. The individual’s beliefs concerning their chronic condition in the form of illness perception have an impact on their health and on the treatment outcomes through active participation in the treatment process [23–25].

Cognitive representation of illness is a fundamental construct included in health behavior models that explain the behaviors resulting from the patient role. Among the numerous models addressing cognitive illness representation, one of the most frequently cited is Leventhal’s Self-Regulatory Model of illness perceptions [26–28], which describes the way in which people interpret illness and cope with it. The model assumes that cognitive representation of illness contributes to adaptation by regulating the patient’s behavior faced with the illness, also influencing the processing of incoming information. It is derived from the model for coping with chronic disease [29]. The structure of illness perception consists of the following factors: identity–illness identification and knowledge about its symptoms, cause–beliefs about the factors responsible for the occurrence of the disease, consequences–the patient’s beliefs about how the disease will affect different areas of their life, timeline–expectations concerning the progress and duration of the disease (whether it is chronic or temporary), coherence–having a coherent and comprehensible illness perception, personal control/treatment control–sense of control over the disease and over the treatment process, and emotional representation–emotional responses towards the disease, sense of anxiety or lowered mood as a result of the condition [30].

Illness perception according to the model by Maes et al. [29] determines the current assessment of the illness situation and the associated behaviors. It is used to explain the psychological and physical consequences of sudden and chronic illnesses [31]. Its utility has been empirically validated in predicting well-being, including psychological well-being, for numerous diseases [31], including rheumatoid [32], lung cancer [33], and ischemic heart disease and associated myocardial [34, 35].

The components of illness perception are significantly related to the process of adaptation to illness–in fact, they determine reactions to the symptoms that appear and their consequences for individual functioning, mainly in the form of assessment of quality of life or emotional functioning. Elements of illness perceptions such as uncertainty about prognosis and symptoms, but also their impact on daily functioning and the ability to control them, are significant for quality of life (mainly psychological), but also for depression and anxiety [3]. If the symptoms of the disease can in themselves affect the health and well-being of patients, a negative illness perception can also result in reduced quality of life. A consequence of this is a number of compensatory strategies in the form of avoidance, over-involvement, or preoccupation with anxiety. Various meta-analyses have shown that perceiving the illness as more severe and having more consequences was associated with worse health outcomes than perceiving it more positively [31, 36]. Research also suggests that illness perceptions can change over time [37–41], nevertheless there is also data suggesting its stability over time ([42]. Living with a chronic condition can gradually change the perception of the associated consequences, emotional representation, and control. Consequently, different trajectories of illness perception over time can be expected in individuals with different types of the illness. Initially, however, illness perception will be related to the immediate emotional response to the diagnosis, and it may therefore be one of the important factors relevant for the assessment of the adaptation process (especially if the illness perception stability hypothesis is accepted).

### Configurations of illness perception components

The literature suggests the formation of specific configurations of illness perception features, as they do not occur in an isolated form [43]. It has been noted that illness features can group together to form patterns, fostering adaptation and health behaviors to a smaller or larger extent [36]. McCorry et al. [42] analyzed the level of psychological functioning of women with breast cancer just after the diagnosis, distinguishing two illness perception configurations. One of them made it possible to predict lower levels of anxiety and depression after six months. The women surveyed reported lower levels of distress when they believed that their breast cancer would not last long and would not be cyclical, when they believed that their treatment would be effective, when they did not believe their cancer to be serious or to have severe consequences for their lives, and when they were unable to identify a clear cause of their disease. Importantly, illness perception remained stable in the respondents throughout the study period, despite the change in disease status. This contrasts with the predictions of the self-regulatory model and has important implications for the timing and type of psychological intervention in this field. Other studies have observed changes in illness perception over time among people with back pain [44] and asthma [45], while longitudinal studies conducted among cancer patients have also shown that illness perception remains stable over time [46].

Livneh, Lott, and Antonak [47] also distinguished patterns of adaptation to chronic illness based on differences in levels of adjustment, coping strategies, depression, anxiety and perceived stress, as well as perceived health control. Patients were characterized by having adaptive, maladaptive and moderately adaptive levels of functioning based on several psychological domains, including the cognitive domain of perceived control. Personal control is not the only cognitive component, so taking into account also other variables related to the perceptions of one’s illness may be important in mediating the relationship between illness characteristics and the assessment of the quality of life in relation to the respective condition. Illness perception is a certain mental filter that influences emotional responses and further actions taken in relation to the disease.

Based on the above results, it seems reasonable to expect that also in the group of patients with multiple sclerosis it will be possible to distinguish illness perception configurations. This study attempts to identify these configurations in patients in the early stages of the disease.

### Illness perception and adaptation to illness

It has been suggested that illness perception may be important in explaining the effects of the adaptation process in relation to biological variables [13]. In determining the effects of the adaptation process, the presence of anxiety, depression and quality of life levels are taken as indicators [48, 49]. Research has shown that elements of illness perception such as uncertainty about prognosis and symptoms, but also their impact on daily functioning and the possibility of controlling them, are important for quality of life (mainly psychological) but also for depression and anxiety [3]. Identifying the structure of illness perception makes it possible to conjecture about the potential risk of states of depression or anxiety, or reduced quality of life in general. High levels of depression and anxiety may lead to worse cooperation with the physician, to the emergence of risky behaviors or a reduction in health behaviors, chronic inflammation of the body due to chronic tension or suicidal thoughts/attempts (Patten, [22]. It seems important, therefore, to investigate whether differences can be observed in adaptation indicators in the illness perception configurations distinguished.

### Dynamics of change of adaptation indicators in individuals diagnosed with MS

It seems that the observations from cross-sectional studies concerning higher levels of depression and anxiety in a group of people with multiple sclerosis do not make it possible to draw conclusions about the possible factors that might explain the variation in their levels or predict their future values. Depressiveness and anxiety, on the one hand, intensify with time and as the disease progresses [50], but they can also be lower than in the initial phases of the disease as a result of the adaptation process [51, 52].

Research suggests that depression may be unrelated to disease severity [53, 54]. Additionally, it may be associated with unpredictability and unpredictable prognosis in MS [53]. Lower depression levels have also been observed in individuals with progressive MS compared to those with relapsing MS [55]. On the other hand, the level of anxiety peaks after learning the diagnosis and decrease over time–it is considered a predictor of the adaptation process [56]. Researchers argue that the uncertainty accompanying the course of the disease is important (in a progressive course, patients expect gradual loss of function, whereas in a relapsing-remitting course, symptoms intensify or diminish in a very intrusive manner) [3, 57]. Consequently, it can be assumed that the role of depressiveness and anxiety will depend on illness perception. Zhang et al. [58], in their study of neck and head cancer, identified a negative illness perception as burdened by more symptoms of the illness, with significant impact on functioning, longer duration and less control over its treatment and course. In that study, perceived symptoms of illness were a predictor of the level of psychological distress, i.e. of high levels of anxiety and depression. One may therefore ponder on the potential differences in the relationships between depression, anxiety and quality of life depending on the patient’s illness perception–it can be expected that in the case of adaptive illness perception, with fewer perceived symptoms associated with MS, lower levels of depressiveness and anxiety will predict lower values of these variables in the future, while at the same time these values in the respective group will be lower, and the relationship will be stronger.

According to Bassi et al. [59], illness perception characterized by illness coherence and high levels of personal control and treatment control will be associated with better adjustment, while high levels of negative emotions and the belief that the symptoms are cyclical will be associated with worse adjustment. Moreover, the way of perceiving the symptoms can change the direction of the adaptation process [41, 60]. This has also been demonstrated in various diseases such as heart conditions [61]. It has been found that attitudes towards the illness and the accompanying emotions increased or decreased depression, anxiety, and quality of life. This affected the nature of the adjustment and recovery process. Therefore, it can be assumed that the distinguished configurations of illness perception that differ in terms of quality of adaptation will favor its different directions, and therefore different levels of depression, anxiety, and quality of life.

### Current study

The main aim of the current study is to assess the role of illness perception in adaptation to multiple sclerosis. The specific objective of the study is to assess the levels of depressiveness, anxiety, and illness-related quality of life among patients with multiple sclerosis and to assess the possibility of predicting their levels depending on the nature of illness perception. A longitudinal design was used in this research. Using longitudinal studies will make it possible to assess the importance of illness perception in evaluating the risk of mental disorders as symptoms of adaptation difficulties and the level of quality of life. The development of depressive and anxiety disorders has an adverse impact on the course of the illness, the treatment, as well as the patient’s overall functioning. This in turn leads to questions as to whether illness perception will group together patients with multiple sclerosis on the basis of its components and their configuration.

It is expected, on the basis of the results of the studies described above, that it will be possible to distinguish three types of illness perception: adaptive, moderately adaptive, and maladaptive (Hypothesis 1). It is presumed that an adaptive illness perception will be associated with fewer perceived symptoms, less intense chronicity beliefs, a higher sense personal control/treatment control, and less intense negative emotions. Moderately adaptive and maladaptive illness perception types will be characterized, accordingly, by a higher number of symptoms and a stronger belief about chronicity and severe consequences, lower personal control, and higher levels of negative emotions. The value of distinguishing specific illness perception patterns consists in the possibility of adapting psychoeducation concerning the illness to the individual’s specific beliefs about their condition. This approach is also consistent with the theoretical framework of illness representation, where illness components tend to combine to form representations of the overall perception of the illness.

When distinguishing the different configurations of illness perception, it seems important to check whether they differ in terms of the indicators of the adaptation process. It is also predicted that the group of individuals with an adaptive illness perception will present lower levels of anxiety and depression and higher levels of quality of life than those with moderately adaptive and maladaptive illness perception (Hypothesis 2)

Finally, given the longitudinal nature of the research, it seems reasonable to check whether and how adaptation indicators, i.e. depression, anxiety, and quality of life during the successive measurements, may be related to one another in the consecutive measurements, making it possible to predict their values over time. The direction of these relationships will differentiate the individual configurations. It is assumed that depressiveness, anxiety, and quality of life at T1 are predictors for the same variables at T2, at T2 they are predictors for T3, and at T3 they are predictors for T4. The prediction values are higher in adaptive illness perception (Hypothesis 3).

## Materials & methods

### Study design

The study consisted of four measurements, during which the study subjects were given a set of questionnaires. During the first meeting, as part of a routine medical examination, a neurologist conducted a personal questionnaire and performed a cognitive function test. In the main part of the study, respondents completed the following questionnaires: Illness Representation Questionnaire–Revised (IPQ-R), the Hospital Anxiety and Depression Scale (HADS) to assess depression and anxiety levels, and MSIS-29 to assess quality of life. The measurements were repeated three times (T2, T3, T4), except for the first measure (T1). The intervals between T1, T2 and T3 were three months, and between T3 and T4 they were four years (the disproportionate time between the measurements resulted from the possibilities of collecting them over such a period). The intervals fixed between the measurements resulted from the schedule of outpatient care appointments for patients with multiple sclerosis, the final measurement resulted from the possibilities the researcher had and from the extended interval between the successive outpatient appointments. Two patients did not show up for the second appointment, six patients for the third one, and 11 patients for the fourth one. This represents <3% of the whole group at the second meeting, 6% at the third meeting, and 12% of the patients at the fourth meeting, respectively. Missing data were managed using the multiple imputation method as recommended by Kang [62]. In order to deal with missing data, a multiple substitution technique was applied in SPSS, indicating the variables where data were missing. All data gaps were analyzed. Estimated data gaps were as follows: 16.9% in the first measurement, 18.5% in the second, 22.2% in the third, and 26.9% in the fourth measurement. First, the systems of all missing values of the variables were analyzed and then, through substitution models (five imputations), the gaps were filled using regression analysis.

### Participants

Patients were qualified for the study at a neurology ward or a neurology outpatient clinic initially by the researcher and by a team of physicians working at the respective facilities. The final group, N = 90 of the 425 subjects in total, 44 women (48.89%) and 46 men (51.11%), was selected on the basis of the following criteria: diagnosis of relapsing-remitting multiple sclerosis (the most frequently diagnosed form of multiple sclerosis, especially in the early stages of the disease and at the same time demanding for the adaptation process due to alternating periods of remission and relapses), maximum four years after diagnosis [7], EDSS disability level ≤ 4, no cognitive impairment (MoCA >26,), no relapse in the last three months from the start of the study. The Expanded Disability Status Scale (EDSS) ranges from 0.0 (no neurological symptoms) to 10.0 (death due to the disease). The scale is based on the patient’s physical abilities and deficits within various functional systems. The higher the scale score, the greater the disability. A score of 4.0 means the patient is fully mobile without aid, self-sufficient, able to function for more than 12 hours a day despite severe difficulties related to particular symptoms, and able to walk without assistance and rest for about 500 meters. The current study assumes that 4.0 is equivalent to preserved daily functioning–thus objectively minimizing the occurrence of low quality of life in the physical aspect. In the case of antidepressant treatment initiated before entering the study, patients were consulted by a psychiatrist to determine the advisability of the treatment. In each case, the psychiatrist determined the absence of co-occurring depressive disorders, and the patients’ treatment was initiated before the diagnosis of multiple sclerosis for the following reasons declared by the patients: crisis triggered by another event, low level of behavioral activation, and psychomotor retardation (in the last two cases, these were most probably symptoms of multiple sclerosis).

All patients gave their written informed consent. The study was approved in the first stage by the committee opening the doctoral dissertation procedure (2016), the follow-up was subject to a separate assessment by the university’s local ethics committee and obtained approval (IRB No. 7.2019). 33% of the respondents had completed secondary school, 48% had higher education, and 15% had primary education. The minimum time from the onset of first symptoms was 12 months and the maximum time was 132 months. The minimum number of relapses among the study subjects was one and the maximum number was seven. Among the study groups, 62% (56 respondents) were participants of drug programs. Eight individuals were treated with antidepressants, accounting for less than 9% of the study group. The average level of disability over the years–Kurtzke EDSS for the study subjects was M = 2.4 (SD = 0.8).

### Measures

The Illness Perception Questionnaire–Revised (IPQ-R; [30]. The IPQ-R contains the identity subscale with 14 symptoms often present during illness (e.g. pain, headache and dizziness), the seven subscales (a total of 38 items) assessing timeline acute/chronic (“My illness will last a short time”), consequences (“My illness does not have much effect on my life”), personal control (“What I do can determine whether my illness gets better or worse”), treatment control (“My treatment will be effective in curing my illness”), coherence (“My illness is a mystery to me”), timeline cyclical (“My illness is very unpredictable”), emotional representations (“Having this illness makes me feel anxious”) and the cause scale (e.g. stress, worry, germ, overwork). Items in the main section are rated on the original 5-point Likert type scale: strongly disagree, disagree, neither agree nor disagree, agree, and strongly agree. The third Cause section was not included in the study due to the high percentage of omissions. The reliability of the Polish version conducted for the purposes of the study measured by the alpha coefficient was Identity = .81, Timeline Chronic .60, Timeline Cyclical = .60, Consequences = .75, Personal Control = .59, Treatment Control .86, Illness Coherence .92, and Emotional Representation = .70.

The Hospital Anxiety and Depression Scale (HADS) was constructed by Zigmond and Snaith [63]. The scale assesses the intensity of subjective depression and anxiety in the last week. The Polish adaptation was performed by de Walden-Gałuszko, Majkowicz, and Chojnacka-Szawłowska [64]. It consists of the Anxiety Scale and the Depression Scale. The answers are assessed on a 4-point Likert-type scale–the higher the number of points, the greater the severity of psychopathological symptoms. The reliability of the Polish version conducted for the purposes of the study measured by the alpha coefficient was .87 for the Depression Scale and .76 for the Anxiety Scale.

The Multiple Sclerosis Impact Scale (MSIS-29; [65]) is the scale assessing the impact of MS on the quality of life of the subjects in the physical and mental dimensions (Polish adaptation by Jamroz-Wiśniewska et al. [66]). It consists of 29 questions, of which the first 20 are related to the physical status, and the other questions to the mental state of respondents. The range of the results is from 0 to 100 points. The higher the score, the worse the quality of life. In the study, the scale alpha reliability was .92 for the physical dimension subscale and .94 for the psychological dimension subscale.

### Statistical analysis

Descriptive statistics were calculated for demographic variables, depression and anxiety scales from the HADS questionnaire and quality of life variables from the MSIS-29 questionnaire (general, physical and psychological). The decision was made to choose two dimensions of quality of life due to the possibility of obtaining specific findings compared to the overall level of quality of life. Basic *r*-Pearson correlations were performed. Illness perception configurations were distinguished using cluster analysis with the *k*-means clustering algorithm–the best fit was obtained with three clusters (interpreted respectively as AIP–anxious illness perception, RIP–realistic illness perception, and FIP–fatalistic illness perception). Subsequently, the mean values of depression, anxiety, physical and psychological quality of life were compared between the individual clusters using the Kruskall-Wallis test.

Finally, a cross-lagged panel modeled for HADS and MSIS-29 subscales in each measurement occasion (T1–T4). All variables were modeled as observed variables with error terms included to adjust for external factors that may contribute to observed effects. Model fit was assessed using the Comparative Fit Index (CFI), Tucker-Lewis Index (TLI), Root Mean Square Error of Approximation (RMSEA) with 90% CI and *χ*^*2*^. A good fit was recognized when CFI and TLI were higher than 0.90 and RMSEA and SRMR was close or below .08 and *χ*^*2*^/*df* of 2.00 or below (Kline, 2005). Model parameters were then compared between AIP, RIP and FIP groups allowing for comparisons of relations of anxiety, depression and physical and psychological quality of life in time depending on profile of illness perception.

Analyses were conducted in IBM SPSS Statistics 26 (IBM, 2018) and Mplus 7 (Muthén, Muthén, 2012).

## Results

The descriptive statistics are included in Tables 1 and 2.

**Table 1:** Demographic and disease characteristics.

	M	Min	Max	SD
Age	29.50	19	50	8.26
EDSS	2.41	1	4	0.84
Number of months from first symptom onset	27.72	12	132	24.12
Number of months from diagnosis	24.90	12	100	15.04
Number of months from last relapse	13.67	3	24	5.87
Number of relapses	1.47	1	7	1.17

**Table 2:** The IPQ-R, HADS and MSIS-29 descriptive statistics.

	N	M	Min	Max	SD
Symptoms	90	9.42	2	19	4.29
Timeline chronic	90	21.18	12	26	3.33
Timeline cyclical	90	13.94	10	17	2.11
Consequences	90	19.94	11	27	4.30
Personal control	90	19.14	13	25	2.60
Illness coherence	90	15.26	11	21	2.85
Treatment control	90	16.96	11	22	3.66
Emotional representation	90	21.38	15	28	3.11
Depression (T1)	90	4.62	0	9	2.42
Depression (T2)	88	4.23	0	12	2.48
Depression (T3)	84	4.02	0	10	2.36
Depression (T4)	79	2.87	0	8	1.74
Anxiety (T1)	90	5.50	2	10	2.47
Anxiety (T2)	88	4.23	1	9	1.94
Anxiety (T3)	84	4.22	1	11	2.21
Anxiety (T4)	79	2.68	1	8	1.33
Quality of life (T1)	90	70.70	29	128	24.69
Quality of life (T2)	88	64.80	26	120	24.74
Quality of life (T3)	84	61.76	25	119	26.65
Quality of life (T4)	79	51.04	29	135	23.57
Physical QoL (T1)	90	50.28	20	97	18.40
Physical QoL (T2)	88	47.26	20	93	19.75
Physical QoL (T3)	84	45.48	20	89	21.15
Physical QoL (T4)	79	37.25	20	92	18.82
Psychological QoL (T1)	90	19.68	9	39	8.08
Psychological QoL (T2)	88	17.61	9	31	6.56
Psychological QoL (T3)	84	16.39	9	33	7.02
Psychological QoL (T4)	79	13.78	9	45	6.49

As part of preliminary data analysis, *r*-Pearson correlations were performed: (1) between the illness perception subscales and medical variables, (2) between the illness perception subscales and adaptation indicators in each measure. A negative correlation was observed between age and perception of MS as a chronic disease (*r* = -.335), as well as between age and the perception of chronicity of the disease (*r* = -.232, *p* < .05). Furthermore, a positive association was observed between the number of relapses in general and the perception of MS as cyclical (*r* = .239). When analyzing adaptive indicators, the perception of MS as chronic was negatively associated with anxiety (for T1-4 *r* between .243 and .367), depression (for T1-3 *r* between .255 and .410), and quality of life (for T1-3 *r* between .228 and .275). Similar results were found for illness coherence and treatment control. Emotional representation was positively associated with quality of life results. The results are presented in Tables 3 and 4.

**Table 3:** Pearson correlation coefficients between IPQ-R subscales, age and medical characteristics of MS.

	SYM	T–CH	T–CY	PC	CONS	COH	TC	ER
Age	.150	-.335*	-.162	-.202	-.109	-.192	-.232*	.078
Months from the first symptom	.163	-.136	-.194	.005	.113	-.093	-.077	-.065
Months from the diagnosis	.086	-.024	-.100	.020	.152	-.104	-.126	.001
Months from the first relapse– 1^st^ measure	-.026	-.138	-.067	-.153	.239*	-.169	-.026	.014
Months from the last relapse– 4^th^ measure	-.077	.065	.077	.179	.057	.056	.193	-.024
Number of relapses– 1^st^ measure	-.003	.010	-.218*	.168	.025	-.274*	-.153	.164
Number of relapses– 4^th^ measure	-.004	-.102	.115	-.144	.046	-.060	.063	.002
Number of relapses through the disease	.133	.060	.235*	.146	-.039	.155	.096	-.111
EDSS– 1^st^ measure	.111	.030	.056	-.060	.200	.186	.200	-.114
EDSS– 4^th^ measure	.148	-.054	.142	-.054	.176	.210	.184	-.179

*p < .05

Legend: SYM–symptoms, T-CH–timeline-chronic, T-CY–timeline-cyclical, PC–personal control, CONS–consequences, COH–coherence, TC–treatment control, ER–emotional representation.

**Table 4:** Pearson correlation coefficients between IPQ-R subscales, HADS (depression and anxiety) and MSIS-29 with subscales (quality of life, physical QoL and psychological QoL).

	SYM	T–CH	T–CY	PC	CONS	COH	TC	ER
Depression (T1)	.072	-.236*	-.186	-.110	-.121	-.273*	-.185	.063
Depression (T2)	.080	-.410*	-.402*	-.202	-.145	-.445*	-.435*	.122
Depression (T3)	.125	-.382*	-.357*	-.207	-.181	-.449*	-.421*	.215
Depression (T4)	.102	-.255*	-.341*	-.034	-.216	-.360*	-.380*	.221
Anxiety (T1)	.108	-.248*	-.121	-.032	.070	-.121	-.046	.045
Anxiety (T2)	.155	-.243*	-.147	-.104	,071	-.272*	-.224*	.138
Anxiety (T3)	.082	-.367*	-.195	-.272*	.061	-.310*	-.217	.113
Anxiety (T4)	-.076	-.219	-.106	-.230*	-.008	-.269*	-.248*	.259*
Quality of life (T1)	.275*	-.190	-.142	-.041	.088	-.179	-.001	.107
Quality of life (T2)	.235*	-.338*	-.209	-.195	-.029	-.343*	-.163	.244*
Quality of life (T3)	.228*	-.387*	-.306*	-.213	-.081	-.448*	-.264*	.333*
Quality of life (T4)	.108	-.297*	-.282*	-.010	-.171	-.447*	-.300*	.418*
Physical QoL (T1)	.246*	-.145	-.164	-.005	.077	-.190	-.012	.114
Physical QoL (T2)	.202	-.322*	-.190	-.206	-.008	-.314*	-.143	.218
Physical QoL (T3)	.234*	-.360*	-.308*	-.188	-.068	-.426*	-.236*	.334*
Physical QoL (T4)	.118	-.344*	-.303*	-.132	-.169	-.432*	-.289*	.395*
Psychological QoL (T1)	.335*	-.240*	-.173	-.070	-.019	-.239*	-.058	.159
Psychological QoL (T2)	.275*	-.301*	-.213	-.117	-.083	-.343*	-.190	.257*
Psychological QoL (T3)	.154	-.382*	-.228*	-.247*	-.097	-.412*	-.293*	.253*
Psychological QoL (T4)	.051	-.080	-.148	.019	-.130	-.370*	-.251*	.373*

*p<0.05.

Legend: SYM–symptoms, T-CH–timeline-chronic, T-CY–timeline-cyclical, PC–personal control, CONS–consequences, COH–coherence, TC–treatment control, ER–emotional representation.

**Fig 1:**
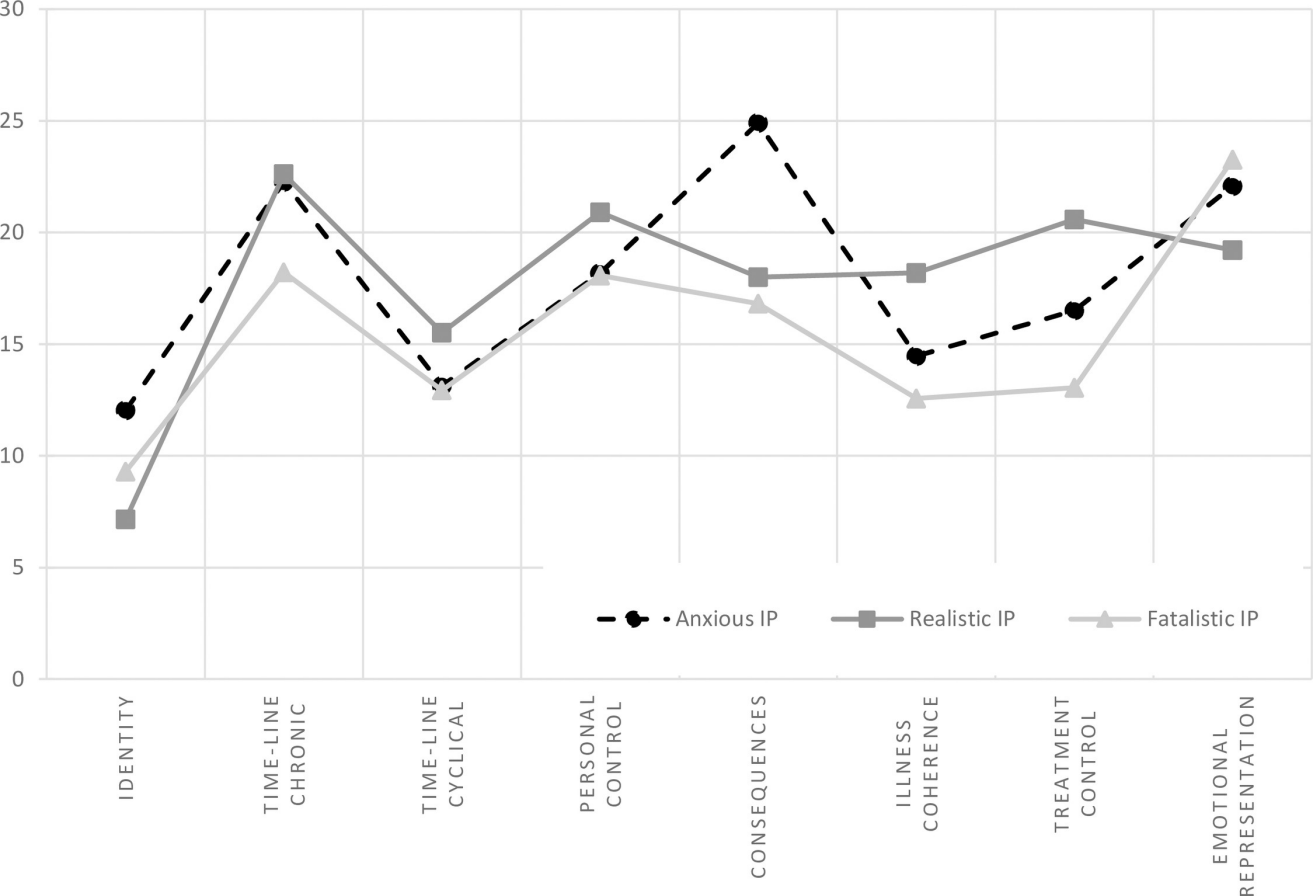
Standard means of clusters distinguished in cluster analysis for illness representation in T1 AIP (*n* = 30), RIP (*n* = 33) and FIP (*n* = 27).

Cluster analysis with the *k-*means clustering algorithm was performed to distinguish the illness perception configurations. The analysis revealed three clusters, defined as Anxious Illness Perception (AIP), Realistic Illness Perception (RIP), and Fatalistic Illness Perception (FIP). Other solutions suggesting the presence of fewer or more clusters demonstrated worse fit. The system of variables and clusters is shown in Fig 1. Table 5 shows the mean values of the variables for each cluster and the results of the variance analysis, carried out to assess the significance of the variability of the results within the identified clusters. Its results indicate the significance of the differences between clusters (p < .001) for all components.

**Table 5:** Mean values of illness perception components in the individual clusters.

Illness representation	Anxious IP	SD	Realistic IP	SD	Fatalistic IP	SD	F (2.87)	p
Identity	12.033	4.567	7.152	3.183	9.296	3.593	12.900	< .001
Timeline chronic	22.267	3.269	22.606	2645	18.222	2.082	22.796	< .001
Timeline cyclical	13.133	2.193	15.515	1.302	12.926	1.662	21.157	< .001
Personal control	18.167	2.151	20.909	2.127	18.074	2.464	16.173	< .001
Consequences	24.900	1.936	18.000	3.335	16.815	1.415	94.778	< .001
Illness coherence	14.467	1.852	18.182	1.236	12.556	1.695	97.141	< .001
Treatment control	16.500	2.838	20.576	1.347	13.037	1.372	109.004	< .001
Emotional representation	22.067	2.815	19.212	1.453	23.259	3.381	19.358	< .001

Cluster 1. Anxious Illness Perception (30 patients)–patients from this cluster scored high on perceived symptoms, and negative consequences of illness. They obtained moderate scores on personal control and illness coherence. They scored high on the level of negative emotions.

Cluster 2. Realistic Illness Perception (33 patients)–patients in the second cluster presented a realistic illness perception, involving low levels of perceived symptoms and high levels of belief in the cyclical nature of the disease. This group displayed high levels of personal control over MS and higher levels of illness coherence compared to other patients. These patients presented low levels of negative emotions.

Cluster 3. Fatalistic Illness Perception (27 patients)–the third cluster of cognitive representations demonstrated the highest level of negative emotions related to illness perception. The patients displayed low levels of perceiving the disease as chronic or cyclical, low levels of negative consequences, sense of control over the disease and sense of coherence in terms of knowledge and information about MS.

The Kruskal-Wallis test with post-hoc comparisons was conducted to compare the mean values of quality of life, depression and anxiety measures in the individual clusters (Table 6).

**Table 6:** Comparisons of aspects of adaptation between AIP, RIP and FIP clusters.

	Timepoint	Cluster	Mean Rank	Kruskal-Wallis (*p*)	Post-hoc (*p*)	
					AIP	RIP
Depression	T1	AIP	42.42	.032		
		RIP	39.53			
		FIP	56.22		< .05	< .05
	T2	AIP	40.10	< .001		
		RIP	34.52		< .05	
		FIP	62.08		< .05	< .05
	T3	AIP	40.48	< .001		
		RIP	33.42		< .05	
		FIP	57.67		< .05	< .05
	T4	AIP	37.32	< .05		
		RIP	34.76			
		FIP	49.98		< .05	< .05
Anxiety	T1	AIP	43.08	.517		
		RIP	43.83			
		FIP	50.22			
	T2	AIP	42.53	.087		
		RIP	39.23			
		FIP	53.39			
	T3	AIP	38.95	.011		
		RIP	37.49		< .05	
		FIP	56.02		< .05	< .05
	T4	AIP	37.06	.014		
		RIP	35.58			
		FIP	52.33		< .05	< .05
Physical QoL	T1	AIP	47.20	.231		
		RIP	39.61			
		FIP	50.82			
	T2	AIP	44.33	.026		
		RIP	36.62			
		FIP	54.69		< .05	< .05
	T3	AIP	44.41	< .05		
		RIP	32.72			
		FIP	53.70		< .05	< .05
	T4	AIP	40.46	.012		
		RIP	31.84			
		FIP	50.50		< .05	< .05
Psychological QoL	T1	AIP	44.45	.098		
		RIP	39.52			
		FIP	53.98			
	T2	AIP	41.33	.010		
		RIP	37.46			
		FIP	56.98		< .05	< .05
	T3	AIP	39.55	< .05		
		RIP	35.72			
		FIP	55.65		< .05	< .05
	T4	AIP	40.74	< .05		
		RIP	32.69			
		FIP	49.04		< .05	< .05

Post-hoc comparisons indicated that Fatalistic and Realistic IP differed in every studied area, namely depression (in all timepoints), anxiety (3^rd^ and 4^th^ stage), psychological quality of life (2^nd^ to 4^th^ stage), and physical quality of life (2^nd^ to 4^th^ stage). Differences in the same areas and stages were observed between FIP and AIP. On the other hand, AIP differed from RIP in fewer areas: 2^nd^ and 3^rd^ stage in depression and 3^rd^ and 4^th^ stage in anxiety.

Overall, FIP achieved the lowest scores in adaptational outcomes in most stages of all studied areas, comparing to AIP and RIP. It was shown that FIP scored low on positive health indices and high on negative health indices.

The lack of statistically significant differences between AIP and RIP was noted in quality of life and anxiety (except 3^rd^ stage). This would indicate a similar nature of these clusters–with AIP and RIP potentially based on a real heath threat but still actively coping and confronting with stressors. The positive adjustment indicators were related to the Realistic IP cluster–with higher psychological and physical quality of life connected with MS.

**Fig 2:**
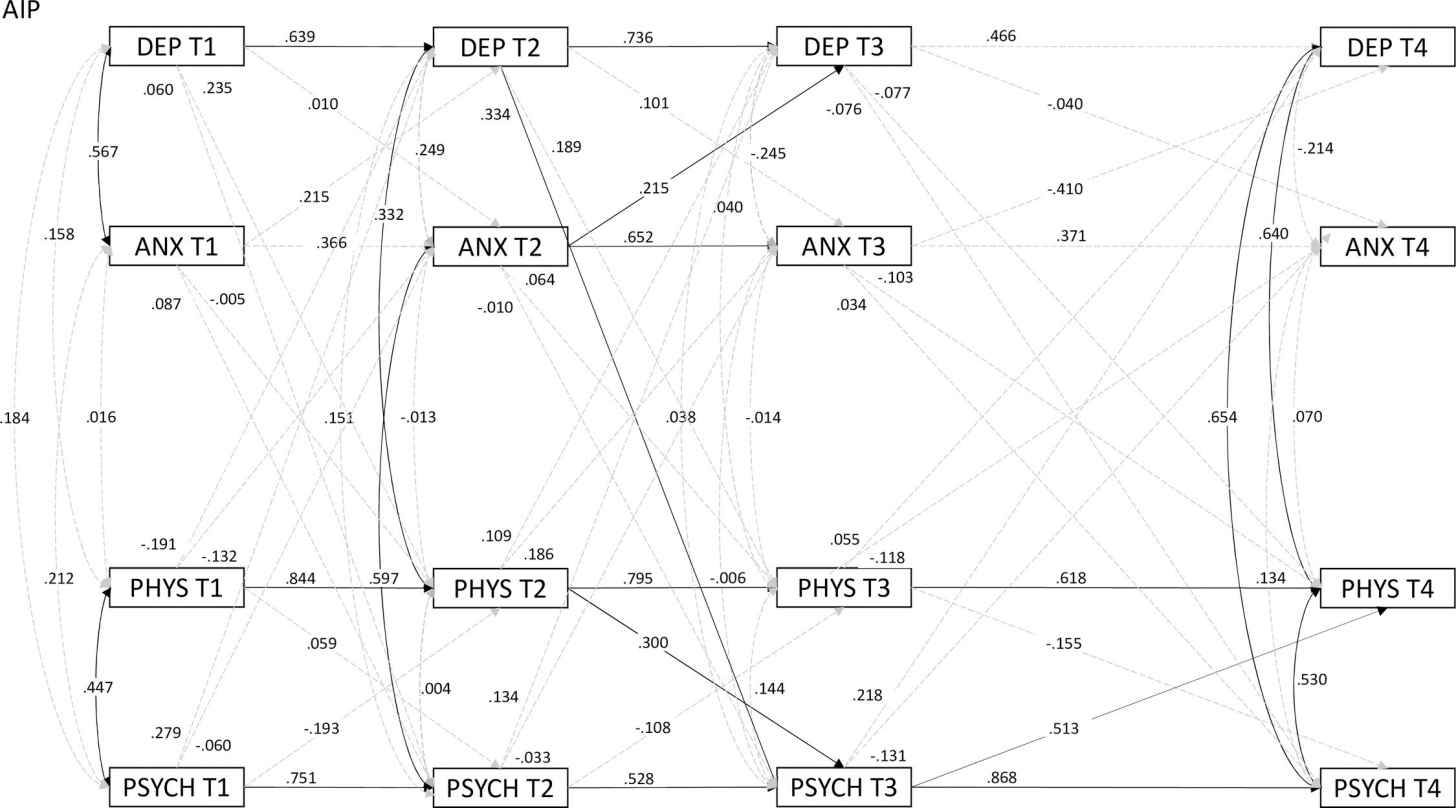
Cross-lagged panel model of MSIS-29 and HADS over four timepoints for AIP (*n* = 30). *Note*: Solid arrows are *p* < .05; dashed arrows are nonsignificant; numbers above arrows indicate standardized coefficients.

**Fig 3:**
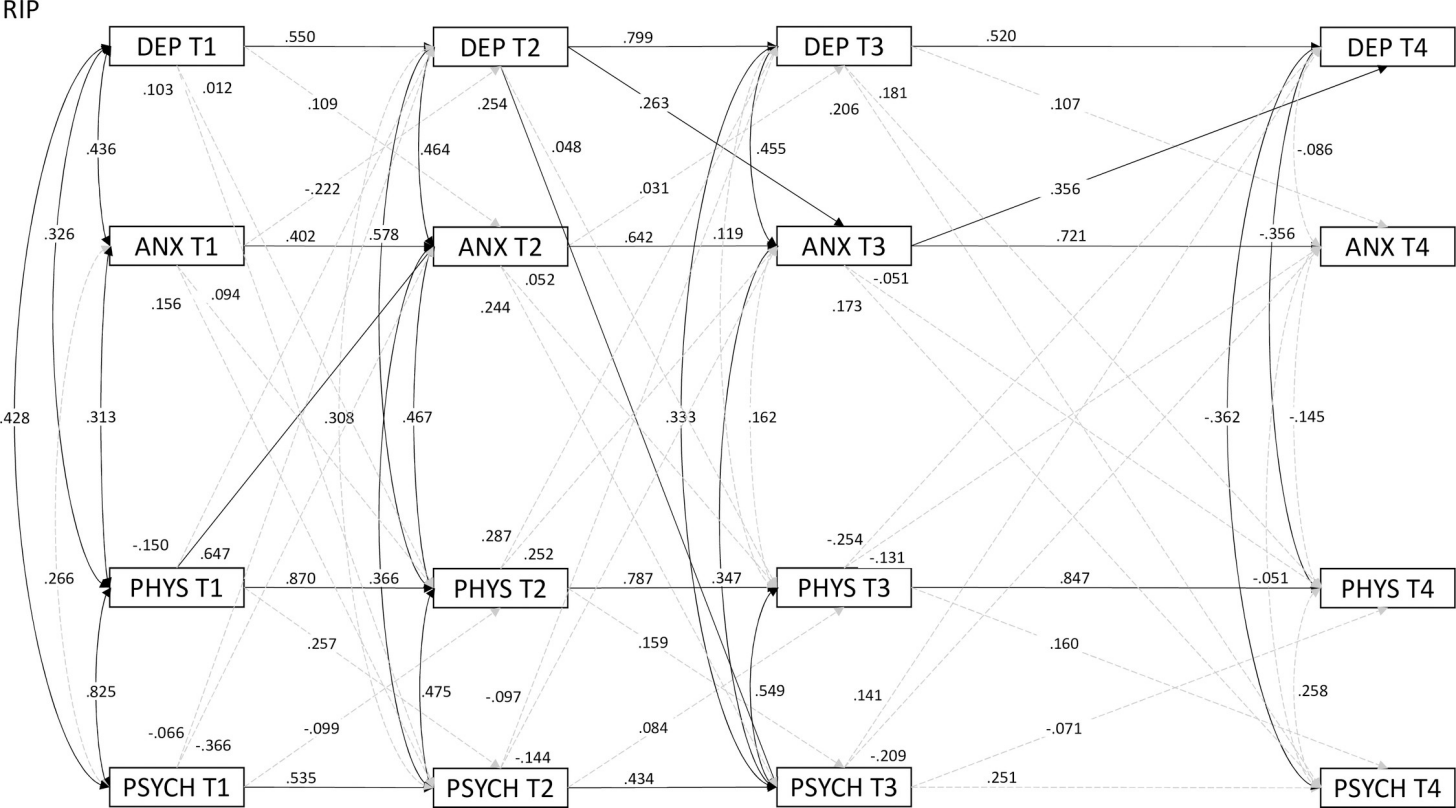
Cross-lagged panel model of MSIS-29 and HADS over four timepoints for RIP (*n* = 33). *Note*: Solid arrows are *p* < .05; dashed arrows are nonsignificant; numbers above arrows indicate standardized coefficients.

**Fig 4:**
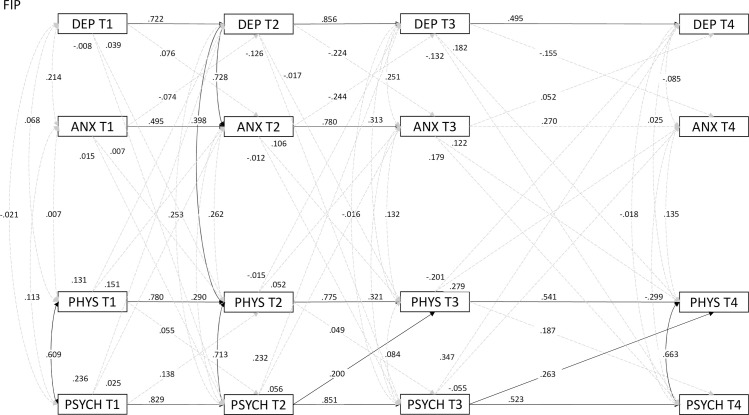
Cross-lagged panel model of MSIS-29 and HADS over four timepoints for FIP (*n* = 27). *Note*: Solid arrows are *p* < .05; dashed arrows are nonsignificant; numbers above arrows indicate standardized coefficients.

In order to analyze the relations of depression, anxiety, and physical and psychological quality of life over time, a cross-lagged panel analysis was conducted on HADS and MSIS-29 scores in each timepoint. The model presented rather satisfactory fit (*χ*^2^(48) = 81.05; CFI = .968; TLI = .925; SRMR = .050) with slightly inflated RMSEA = .087 (90%CI .053-.120). Cluster groups under this model are presented in Figs 2–4.

In RIP, positive predictions of each subsequent measurement by the previous one were observed (except psychological QoL in T3 and T4), as well as positive cross-predictions by previous timepoint, such as physical QoL in T1 predicted anxiety in T2. In AIP, no significant relations were observed between T3 and T4 in anxiety and depression, which can be seen in RIP and FIP. AIP and FIP, compared to RIP, present overall weaker correlations between HADS and MSIS-29 subscales in all timepoints, especially T1. In the last timepoint, FIP presented no correlations between variables, except QoL subscales, and correlations in AIP were positive, as opposed to the negative ones in RIP.

## Discussion

The research has shown that patients with multiple sclerosis differ in terms of their illness perception and components of the latter at the early stage of the disease. The configurations that developed differed in terms of the levels of depression, anxiety, and quality of life depending on the measurement timepoint, accentuating the important role of temporal dynamics in the assessment of adaptation indicators. In addition, the variables analyzed in each measure were significant predictors of the level of other adaptation indicators in subsequent years. This confirms the multifactorial nature of the adaptation process, which, when observed from the moment of diagnosis, makes it possible to implement therapeutic interventions matching to the cognitive representation of the disease. The dynamics of adaptation indicators is evidence of the changes that occur in the coping process, worthy of attention on the part of clinicians.

### Illness perception

It was assumed that it would be possible to distinguish adaptive, moderately adaptive and maladaptive illness perceptions. Hypothesis 1 was confirmed by distinguishing three configurations of illness perception components, named respectively: RIP–realistic illness perception (adaptive), AIP–anxious illness perception (moderately adaptive), and FIP–fatalistic illness perception (maladaptive).

An anxious illness perception was characterized by perceiving the illness as chronic, mainly by focusing on the physical symptoms and perceived negative consequences of the illness (which may be exaggerated and never actually appear). These characteristics significantly differentiated the respective patients from those belonging to the other two groups. At the same time, there was a moderate level of control and negative emotions.

Patients presenting a realistic illness perception had the lowest level of perceived symptoms, most likely corresponding to the situation of relatively low disability and disease progression. They indicated a higher level of personal and treatment control, which was a significant difference compared to the other groups. MS was more comprehensible for these patients, which may explain the higher level of coherence compared to AIP. At the same time, the level of perceived chronicity and cyclicality was moderate. This group also had the lowest levels of negative emotions.

As opposed to the other types of illness perception, the third group of patients, those with fatalistic illness perception (FIP), presented mostly negative aspects of illness representation–low control over the course of the disease and high levels of negative emotions. Patients with FIP were also the oldest participants in the study. They perceived a moderate number of symptoms of the disease, but did not assess the latter as chronic or cyclical–suggesting a resigned attitude or even denial that the illness is real. The patients displayed an attitude in which they did not want to recognize the different characteristics of the disease, trying to function, as it were, in detachment from it. Aspects such as the lowest perceived chronicity or negative consequences suggest that they may present avoidant behaviors that do not confront the reality of the illness.

The above illness perception types are consistent with the findings of studies concerning illness perception in various chronic conditions: chronic pain [67], arterial hypertension [68], type 2 diabetes [69], or early stages of breast cancer [42]. The characteristics differentiating the individual clusters were similar factors obtained in the current study–perceived symptoms, consequences, level of negative emotions, level of control and perception of chronicity of the course of the disease. Patients displaying the highest level of adaptation tried to perceive fewer physical symptoms and fewer direct effects of the disease. At the same time, they declared a higher level of control over the disease, also in the context of the treatment process, and lower levels of depression and anxiety. The consistency of the results suggests that chronic diseases may share a similar cognitive background in terms of how patients perceive and experience their illness.

### Differences in variable levels depending on illness perception configuration

Data were also obtained suggesting significant differences between the profiles in terms of the variables analyzed. Hypothesis two was confirmed. Patients presenting a realistic illness perception scored lower on depression and anxiety compared to the other patients, and perceived significantly fewer limitations and negative effects of the illness over the study period. This is a significant result for the final measure (T4) which persisted after four years and with gradual progression of the disease.

RIP scored significantly highest on psychological quality of life and in the physical domain. This suggests the highest level of adaptation to illness, confirming the findings of the research by Hobro et al. [67] and the results obtained by Graham et al. [70]. Configurations associated with indicators suggesting higher levels of adaptation were associated with realistic views on duration, greater coherence, sense of control, lower levels of negative emotions associated with the illness, and low levels of perceived symptoms (regardless of the disease progression).

In the present study, FIP and AIP subjects obtained similar scores except in a few dimensions. The structure of these representations was similar in terms of symptom perception, cyclicality, control over MS, coherence, and negative emotions. Differences emerged in the perception of the consequences and chronicity–patients in the AIP group carefully identified negative consequences being aware of the chronicity of the condition, while patients in the FIP group displayed the opposite attitude. They focused neither on the consequences nor on the belief in chronicity. As a result, the latter group scored the lowest on adaptation indicators. It seems that perception of consequences can act as a buffer, helping MS patients prepare to confront relapse, just like recognizing the chronicity of the condition. What was described in the research by Kunschitz [71] as an optimistic attitude based on resilience may in fact be an attitude that denies the reality of functioning with a chronic illness, looking at the negative effects of having FIP. The meta-analysis by Rivera et al. [36] suggested that lower levels of perception of consequences may be more adaptive, but the results suggest that it may also lead to adaptive difficulties. Moderate levels of this factor appear to have relatively positive effects on quality of life and emotional functioning.

On the basis of the results of repeated measures, making it possible to look into the adaptation process, it seems possible to conclude that the cognitive profile is related to most adaptation indicators, especially in the second part of the study (T2). Depression, anxiety, and quality of life differed significantly, especially between RIP and FIP. Thus, the suggestion appears that confronting reality and the absence of avoidant coping may lead the patient to seek useful solutions in their daily struggles associated with MS. Non-significant differences between the clusters when measuring depression (T1), anxiety (T1, T2, T3), and quality of life (T1 and T2) in the early stages of the research may be due to the active process of acquiring disease experience. This is particularly the case when the first years of living with MS patients make it possible to gain experience of what the disease looks like, what its course will be like and what possibilities there are of adapting to the difficulties that arise. As found in the literature, patients are less likely to engage in health behaviors or comply with medical advice when their illness perception is negative [39, 72, 73]

The age of the patients also differed between the clusters, with FIP represented by the oldest group of patients. This is consistent with the literature on somatic conditions) [74] and mental conditions [75], with older patients declaring negative illness perception (in cognitive and emotional terms) and lower quality of life compared to younger patients.

### Adaptation process

The study observed different trajectories of the adaptation process for the individual disease perception types. Consequently, the assumptions concerning the third hypothesis were confirmed.

In Anxious Illness Perception (AIP), the level of depressiveness and anxiety was a predictor for subsequent measures in the initial phase of the study. However, it was not involved in explaining their level in the final phase of the study conducted after several years. This is evidence of the possible involvement of other explanatory variables in this process, at the same time showing the absence of a direct relationship between how the patients functioned emotionally in the early period of diagnosis and the later several-year period. Interestingly, depressiveness in the final measure is associated with lower levels of physical and psychological quality of life. This indicates the need to take into account other variables that could significantly explain the patients’ emotional state. Their illness representation is dominated by perceptions of the consequences of the illness and the sense of chronicity, but at the same time there is a moderate level of control favoring action while living with the illness.

An interesting association is also represented by depressiveness in the second measure as a predictor of lower psychological quality of life in the third measure (or the perceived burden of illness). This may suggest the potential value of monitoring the state of depression as contributing to negative psychological functioning in patients, but resulting, as the disease progresses, from causes other than the state of depression itself.

In terms of quality of life (QoL), similar predictive relationships were observed for the physical and psychological dimensions, in this case also with regard to the final measure. Interestingly, the psychological quality of life score on the third measure was a predictor of both physical and psychological QoL, but the quality of life in the physical dimension made it possible to predict only the values of that dimension. This suggests a partial independence of the subjective assessment of physical and mental state. At the same time, quality of life in the case of this configuration of disease features appears to be a better measure of adaptation to multiple sclerosis. This may also be related to the measure used to assess quality of life that was directly related to multiple sclerosis.

Research has confirmed that predictors of depression in multiple sclerosis include general medical status or progressive disability [76, 77]. Predictors of anxiety in the disease discussed here pose a greater challenge for researchers, as the findings are inconsistent and often do not yield significant conclusions [76], while noting that anxiety levels are significantly higher in MS patients than in the general population [78].

The observations obtained may also mean that the subjective assessment of the physical effects of the disease explains at the beginning of the illness how the patient further evaluates their physical, but also their mental condition. However, after four years, physical quality of life is no longer that significant; only the assessment of the psychosocial consequences of the disease determines the overall quality of life. Worrying about the future starts, along with a subjective assessment of the limitations caused by the illness. Anxious illness perception may be characterized by cognitive errors such as tunnel thinking, which may involve focusing on selective aspects of the illness consistent with subjective perception of the latter [79]. This may also imply the potential role of metacognitive beliefs about emotional state, which may significantly alter the way of perceiving and experiencing the illness, as suggested in a group of patients with diabetes [80].

Analyzing covariance between the variables in each measure, it was noted that in the initial stage of the study (T2), higher levels of depressiveness were associated with lower levels of physical quality of life, while higher levels of anxiety were associated with lower levels of psychological quality of life. This may suggest that perceiving negative symptoms of the illness may promote the emergence of a negativistic evaluation of the reality, while a state of anxiety and elevated tension may be associated with imagining the future and the tendency to worry. According to the concept by Moss-Morris [30], the anxiety associated with chronic illness decreases over time, patients become accustomed to their illness and learn to live with it. This is confirmed both by earlier analyses and by the non-significance of predictions at the beginning and at the end of the study. Other research suggests that depression is more important in explaining psychosocial functioning, as it was found to be associated with greater physical disability and lower levels of occupational functioning, while anxiety was not [81].

In Realistic Illness Perception (RIP), anxiety, depressiveness and physical quality of life in each consecutive measure constituted predictors for the subsequent levels. An exception here was psychological quality of life, which was not explained by any of the analyzed variables in the final measure (T4). This suggests, on the one hand, the involvement of other factors responsible for assessing patients’ psychological functioning, but, on the other hand, it highlights the value of monitoring emotional state and of subjective assessment of physical state by the patients. An interesting observation is related to depressiveness at T2, as a positive predictor for anxiety at the subsequent T3 measure. Anxiety, in turn, made it possible to predict depressiveness at T4. In other words, the persistence of anxiety states potentially resulting from the monitoring of the source of threat represented by the illness may, in a longer-term perspective, mask depressive states. Such dynamics may also result from an increasing awareness of physical limitations. This is consistent with research in which depressive states and feelings of fatigue tended to persist, while the level of anxiety initially co-occurred but nevertheless decreased over time and as the disease progressed [82]. Compared to the model for AIP, patients with a realistic illness perception reduced their anxiety levels either more rapidly or more effectively in the process of adaptation to illness. The role of depression in this process can also be reflected upon–it represents a disproportionate emotional response to the patient’s actual physical state. Through this emotional coping, all the patient’s resources are consumed, and instead of coping with the disease in the surrounding reality, the patient focuses on their own emotional state with a self-referential attitude. Research confirms that higher levels of depressiveness are observed during the first years of the disease [53, 54] and may be relatively independent of the patient’s physical condition. In addition, Butler et al. [83] emphasize that anxiety is often overshadowed by depressiveness.

On the other hand, lower levels of depressiveness in the final measure were associated with lower values of both psychological and physical quality of life, suggesting that we may be dealing with an apparent realism that favors negative emotions after several years of living with the illness. Initially, anxiety and depression alternate, occurring from time to time at different points during the course of the disease. Ultimately, this remission of depressive states is associated with a reduction in quality of life, probably through a mechanism of focusing on the reality of living with the illness. Realistic illness perception is characterized by perceiving multiple sclerosis as a chronic and cyclical condition that can remain under control, just like its treatment. Moreover, this representation is not accompanied by a similar level of negative emotions as in the case of an anxious or fatalistic illness perception. This is also consistent with the research by Hanna and Strober [84], in which an interdependence was found between anxiety and depression was observed, particularly among those with shorter illness duration.

Illness coherence is characteristic of this RIP representation. According to the salutogenic model [85], coherence is an important resource for improving resilience to stress and threatening states. It can facilitate adaptation to illness and well-being [86]. The findings of Bassi et al. [59] supported this interpretation: understanding a disease such as MS and accepting its basic features (unknown etiology, highly variable symptoms, and unpredictable course) makes up a global attitude that is positively related to satisfactory quality of life. However, it seems that the process of giving coherence to the experience is lengthy, as the current study suggests. The belief about treatment control present in this illness perception type suggests that even if MS cannot be cured, confidence as to the effectiveness of treatment in controlling disease progression or the symptoms can be a valuable source of control, with positive consequences for the patients’ well-being. A realistic approach to the core symptoms and progression of the disease allows patients to get closer to the essence of the latter and to learn to live with disability, but at the same time avoid looking towards the future. Similar conclusions come from longitudinal studies on patients with esophageal cancer [46]

Among patients with fatalistic illness perception (FIP), depressiveness was a predictor of a subsequent negative emotional state. Quality of life in the physical and psychological dimensions also made it possible to predict subsequent values in further measures. High values of depressive states favored the persistence of such states after several years of living with the illness, just as low quality of life was a predictor for similar values in the course of the illness. Interestingly, as in the case of AIP, anxiety in the T4 measure was not explained by any variable, while at the same time having the highest value in this disease representation.

In addition to similar observations in which the individual variables of anxiety, depressiveness, and quality of life were predictors of the value of subsequent measurements of the same variables–significant relationships of depressiveness with anxiety and physical quality of life were observed initially (T2), but this relationship ceased to be significant after four years. Perhaps decreasing anxiety fostered confrontation with the negative vision of the disease. An interesting relationship in the model is represented by the predictive values of psychological quality of life, to some extent determining physical quality of life. A subjective perception of the negative psychological consequences favored a negative perception of multiple sclerosis symptoms (despite the absence of clear disease progression). It may seem that FIP involves high costs in terms of emotion regulation, which can be reflected in the level of well-being. Bassi et al. [59] demonstrated a relationship in which negative emotional representations were associated with lower psychological well-being, life satisfaction, and emotional balance. This result is consistent with previous research showing that depression had a significant negative effect on HRQoL among patients with multiple sclerosis [12]. The more MS patients tried to distract themselves from the disease, the less they were able to gain insight into specific features of the latter. Fundamental knowledge about the disease will help one to adapt well, but also to return to everyday life–with possibilities and limitations.

One of the possible explanations for the interdependence between depressiveness and anxiety and quality of life dimensions is based on resilience: over time, MS patients may know better what they are dealing with, develop better regulation abilities, and have access to psychological resources that sustain their psychological well-being. Research by Kasser and Zia [87] suggested that resilience might be a protective factor against a decline in patients’ quality of life and well-being despite disease progression. Resilience as such has been repeatedly studied in the aforementioned group [88–90]. It seems that supplementing the future model by adding this construct may contribute more to the understanding of the dynamics of change in the functioning of patients with multiple sclerosis.

In conclusion, it was found significant to perform the final measure at an unequal time interval–among AIP patients, illness-related quality of life was a significant factor characterizing the adaptation process. Among those with RIP, depressiveness, anxiety and quality of life were adaptation predictors, while among those with FIP, only depressiveness made it possible to assess the level of adaptation. Such findings did not appear in previous measures, suggesting the significance of long-term follow-up of patients and of identifying risk factors for the difficulties in adapting to chronic illness. In other words, on the basis of the initial measurements of individual characteristics, it was possible to predict patients’ functioning after several years. The covariance of anxiety and depressiveness was significant for AIP patients, that of depressiveness and anxiety was significant for RIP patients, and that of depressiveness was significant for FIP patients. In addition, each of the variables was a predictor of subsequent measures at particular time intervals, illustrating the dynamics of change.

### Limitations

Inference from the data obtained is subject to limitations for several reasons. Firstly, the method itself and the specific points of measurement–the gap between the third and fourth measurement may have been too large and numerous potential variables may exist affecting adaptation outcomes, such as positive experiences, change in marital status, or change in treatment. It would be useful to include social, personal and emotional resources–in addition to cognitive factors such as illness perception–in the group of variables that may influence the adaptation process. Secondly, the lack of an analysis of confounding variables in the time intervals makes it impossible to conclude on the direct causal determinants of the level of adaptation. The limited sample size also means that conclusions should be drawn with caution. Thirdly, individuals with the relapsing-remitting type of MS were selected for the patient group, with the exclusion of other types of multiple sclerosis. Moreover, the values of the variables may have been underestimated as the group included patients having access to the hospital environment. They were willing to take part in the study, possibly constituting a non-representative group. Fourthly, specific variables related to the status of the disease were not controlled for–health crises may have appeared at individual levels, contributing to the reactive emergence of changes with regard to depressiveness, anxiety, or quality of life.

## Conclusions

Despite the above limitations, a strength of the present study is the fact that it considered the possible trajectories of change in the degree of adaptation to illness of patients with different illness perception types with regard to MS. Patients with RIP displayed lower anxiety and depression levels and higher quality of life compared to AIP and FIP patients. Maintaining a realistic illness perception along with low levels of anxiety and depression made it possible to predict a good level of psychological functioning and higher quality of life compared to the other groups. On the other hand, in the long-term perspective, depressiveness appeared to be significant as an effect of confrontation with barriers caused by the illness. A group at risk of difficulties in the process of adaptation to multiple sclerosis are individuals with a fatalistic or negative perception of the disease. The dynamics of adaptation in this group of patients suggested that they presented depressiveness as a response to living with the illness.

The practical application of the results obtained points to the significance of follow-up in patients with positive/negative illness perception in terms of the unobvious consequences of having such configurations. At the same time, it becomes important to work on reducing anxiety in the early period of the disease, despite the focus on depressiveness in multiple sclerosis that has prevailed so far. Illness perception can be a risk factor for patients’ lack of adaptation to multiple sclerosis, which is why it becomes so important to analyze illness perception after diagnosis, particularly when there is a risk of lack of further contact with the patients. Determining the illness perception type shortly after diagnosis can also help to identify individuals belonging to the group of increased risk of failure to adapt, thus making early psychological intervention possible.

To recapitulate, reference made to the patients’ illness perception by the specialist may foster willingness to engage in medical contact and comply with the recommendations. Previous research has shown that realistic illness perception is recognized correctly as a treatment goal [61, 91], and interventions related to illness perception have shown promising results in patients with chronic diseases [61].
